# Essential Role of Endothelial MCPIP in Vascular Integrity and Post-Ischemic Remodeling

**DOI:** 10.3390/ijms20010172

**Published:** 2019-01-05

**Authors:** Zhuqing Jin, Jianli Niu, Nidhi Kapoor, Jian Liang, Edilu Becerra, Pappachan E. Kolattukudy

**Affiliations:** 1 School of Basic Medicine, Zhejiang Chinese Medical University, Hangzhou 310053, China; jinzq@hotmail.com; 2 Burnett School of Biomedical Sciences, College of Medicine, University of Central Florida, Orlando, FL 32816, USA; Nidhi.Kapoor@ucf.edu (N.K.); Jian.Liang@ucf.edu (J.L.); Edilu.Becerra@ucf.edu (E.B.); pk@ucf.edu (P.E.K.); 3 Office of Human Research, Memorial Cardiac and Vascular Institute, Memorial Healthcare System, Hollywood, FL 33021, USA

**Keywords:** MCP-1-induced protein, endothelial cells, endothelial homeostasis, inflammation, angiogenesis

## Abstract

MCP-1-induced protein (MCPIP, also known as Zc3h12a or Regnase-1), a newly identified suppressor of cytokine signaling, is expressed in endothelial cells (ECs). To investigate the role of endothelial MCPIP in vascular homeostasis and function, we deleted the MCPIP gene specifically in ECs using the Cre-LoxP system. EC-specific MCPIP deletion resulted in systemic inflammation, increased vessel permeability, edema, thrombus formation, and premature death in mice. Serum levels of cytokines, chemokines, and biomarkers of EC dysfunction were significantly elevated in these mice. Upon lipopolysaccharide (LPS) challenge, mice with EC-specific MCPIP depletion were highly susceptible to LPS-induced death. When subjected to ischemia, these mice showed defective post-ischemic angiogenesis and impaired blood flow recovery in hind limb ischemia. In aortic ring cultures, the MCPIP-deficient ECs displayed significantly impaired vessel sprouting and tube elongation. Mechanistically, silencing of MCPIP by small interfering RNAs in cultured ECs enhanced NF-κΒ activity and dysregulated synthesis of microRNAs linked with elevated cytokines and biomarkers of EC dysfunction. Collectively, these results establish that constitutive expression of MCPIP in ECs is essential to maintaining endothelial homeostasis and function by serving as a key negative feedback regulator that keeps the inflammatory signaling suppressed.

## 1. Introduction

Maintenance of vascular integrity is critical for organismal survival. The quiescent endothelial cells (ECs) are not only a barrier between blood flow and the vessel wall, but also actively execute important functions to control fundamental properties such as permeability, blood fluidity, and vasomotor tone [1]. Diverse stimuli in circulation can activate ECs and trigger pro-inflammatory responses that are characterized by increased permeability, induced leukocyte adhesion, and a pro-thrombotic feature. Clinically, uncontrolled activation of endothelium plays a key role in many pathological conditions such as infections, cancer, and cardiovascular diseases [2]. The activation of quiescent ECs to generate the pro-inflammatory response is typically driven by transcription factor nuclear factor κB (NF-κΒ), which not only activates the transcription of the pro-inflammatory genes including TNF-α, interleukin-1(IL-1), E-selectin, vascular cell adhesion molecule 1(VCAM-1), and intercellular adhesion molecule 1(ICAM-1), but also renders ECs more susceptible to apoptosis [3,4,5]. Recently, microRNAs (miRs) have been also identified as a new class of signaling molecules that regulate gene expression at posttranscriptional level and are implicated in endothelial activation [6,7,8]. Thus, identification of key molecules to regain control of endothelial homeostasis has great potential for the development of novel therapeutics.

MCP-1-induced protein (MCPIP, also known as Zc3h12a or Regnase-1), originally discovered as a novel zinc-finger protein induced by monocyte chemoattractant protein-1(MCP-1) [9], was identified as a negative feedback inhibitor of cytokine signaling [10,11,12]. MCPIP not only functions as a suppressor of NF-κB activation [10], but has also been shown to disrupt other inflammatory pathways by regulating mRNA degradation [11], miR synthesis [13] and IL-17 receptor degradation [14]. Expression of MCPIP in ECs is regulated by cytokines [15] and has been observed in murine and human atherosclerotic lesions and rheumatoid arthritis [16]. MCPIP has been shown in cell-based studies to directly regulate key endothelial genes related to cell adhesion and angiogenesis [17]. These findings strongly suggest a crucial role for MCPIP in endothelial homeostasis and function. To investigate the physiological role of endothelial MCPIP in vivo, we deleted MCPIP gene specifically in murine vascular ECs by using the Cre-LoxP system with a mouse strain that specially expressed Cre recombinase in ECs under the control of the vascular endothelial (VE)-cadherin-5 promoter [18]. Analysis of mice with EC-specific MCPIP deletion demonstrated an absolute requirement for MCPIP in control of endothelial quiescence and maintenance of vascular maturation and integrity.

## 2. Results

### 2.1. Endothelial-Specific MCPIP Deletion Leads to Systemic Inflammation and Growth Retardation

**Figure 1:**
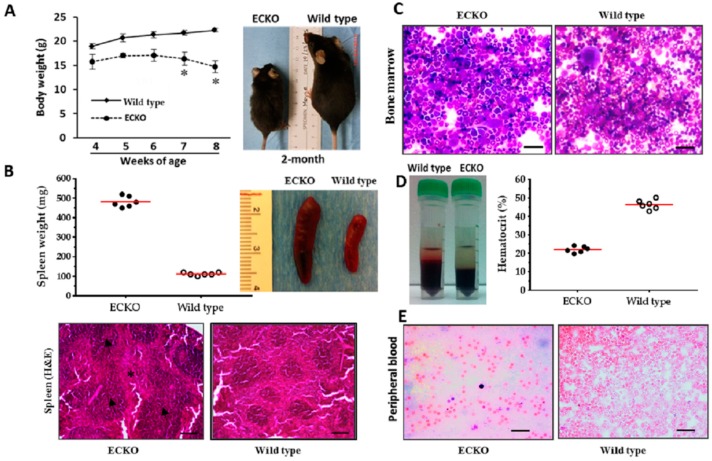
EC-specific MCPIP deletion leads to systemic inflammation and growth retardation. (**A**) Comparison of body weight with age- and sex-matched wild type mice, the ECKO mice showed significantly lower body weight at around two months of age; * *p* < 0.05, *n* = 6. (**B**) Comparison of spleen weight with age- and sex-matched wild type mice, the ECKO mice showed significantly increased spleen weight (*p* < 0.05) and pronounced splenomegaly at around two months of age. Red line represents the mean weight of six individual spleens. *n* = 6 animals per group. Representative H&E stained spleens from the two-month-old ECKO mice showed disrupted architecture containing significantly enlarged white pulps (*arrows*) and red pulps (*star*). Scale bar, 25 µm. (**C**) Bone marrow morphology assessed by Giemsa staining showed decreased erythropoiesis with relatively increased ratio of myeloid to erythroid lineages in the two-month-old ECKO mice. Scale bar, 25 µm. (**D**,**E**) The percentage of hematocrit and numbers of red blood cells in the peripheral blood smear of the two-month-old ECKO mice was markedly decreased compared with the age-matched wild type mice (*p* < 0.05). Red line represents the mean hematocrit of six individual mouse, *n* = 6 animals per group. Scale bar, 25 µm.

To ablate MCPIP gene in ECs, homozygous floxed MCPIP mice [19] were crossed with the VE-cadherin-5-Cre mouse line (Jackson Laboratory, Bar Harbor, ME, USA, Stock #017968), and the progeny were bred to produce mice that are homozygous for EC-specific knockout of MCPIP (ECKO). ECKO mice on a C57BL/6 background were phenotypically normal at birth and nearly indistinguishable during the first three weeks of life. Thereafter, their growth was slower than their littermate controls. The adult ECKO animals were significantly smaller compared to age-matched wild type littermates (Figure 1A). They survived for about 10–14 weeks. To gain insight into the potential causes of these phenotypic differences, gross necropsies were performed to determine the size and weight of major organs. By comparing body weights and spleen weights with age- and sex-matched wild type mice, significantly lower body weight and pronounced splenomegaly were observed in the ECKO mice at around two months of age (Figure 1A). H&E stained ECKO spleens showed disrupted architecture containing significantly enlarged white pulps and red pulps (Figure 1B), indicating splenic hematopoiesis. Because the extramedullary hematopoiesis is likely to be a compensatory mechanism due to bone marrow dysfunction, Giemsa staining was performed to examine the bone marrows of wild type and ECKO mice. ECKO mice exhibited decreased erythropoiesis with a relatively increased ratio of myeloid to erythroid lineages (Figure 1C). Consistently, the hematocrit percentage was markedly decreased in ECKO mice compared with wild type mice at two months of age (Figure 1D). This difference was also reflected in the peripheral blood smear showing markedly reduced numbers of red blood cells in ECKO mice compared with wild type controls (Figure 1E).

We performed multiple analytic profiling on serum from wild type and ECKO mice at two months of age, when showing splenomegaly, to investigate the impact of MCPIP deletion on EC activation. Circulating levels of cytokines, chemokines, and biomarkers of endothelial dysfunction were measured. Of the 32 serum proteins tested, 28 were detectable in the ECKO mice and the age- and sex-matched wild type controls. The ECKO mice showed elevated basal levels of IL-2, IL-3, IL-4, IL-5, IL-6, IL-7, IL-12p40, IL-13, IL-15, IL-17, TNF-α, G-CSF, CCL2, CCL11, MIP-1α, MIP-1β, and CXCL9 in comparison with the wild type controls (Table 1). Of the other analytes tested, vascular endothelial growth factor (VEGF), KC/CXCL1, LIF and IP-10 were elevated while LIX/CXCL5 was decreased in the ECKO mice in comparison with the wild type controls (Table 1). Increased basal levels of soluble adhesion molecules such as sP-selectin and plasminogen activator inhibitor-1 (PAI-1), biomarkers of endothelial dysfunction, were also observed in the ECKO mice (Table 1). Taken together, these results indicate that EC-specific MCPIP deletion induces uncontrolled activation of endothelial, leading to an exaggerated systemic inflammatory response, splenomegaly, growth retardation and premature death in mice.

Results are expressed as mean ± SD (*n* = 3 per group). *p* values are shown for comparison with corresponding wild type controls. G-CSF, granulocyte-colony stimulating factor; M-CDF, macrophage colony-stimulating factor; IL, interleukin; TNF-α, tumor necrosis factor-alpha; CCL, C-C motif chemokine ligand; MIP, macrophage inflammatory protein; CXCL, C-X-C motif ligand; RANTES, regulated on activation normal T cell expressed and secreted; KC, keratinocyte chemoattractant; LIX, C-X-C motif ligand 5; IP-10, interferon gamma-induced protein 10; LIE, leukemia inhibitory factor; VEGF, vascular endothelial growth factor; PAI-1, plasminogen activator inhibitor-1; sP-selectin, soluble platelet selectin.

### 2.2. Endothelial-Specific MCPIP Deletion Leads to Vascular Leak and Systemic Coagulopathy

**Figure 2:**
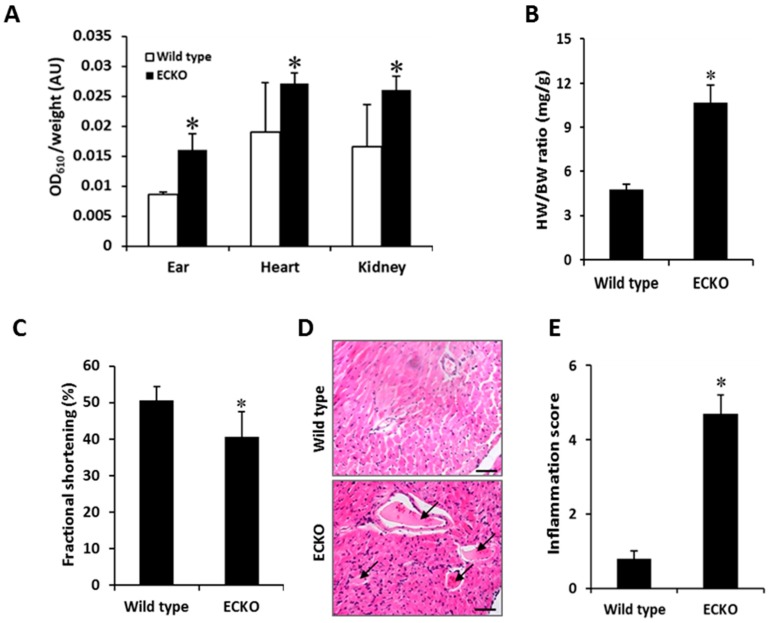
EC-specific MCPIP deletion enhances vascular permeability, cellular infiltration and vascular thrombosis. (**A**) Vascular permeability assay in vivo by injection of Evans blue dye showed increased dye retention in the ear, heart, and kidney tissues from the two-month-old ECKO mice, indicating increased vessel permeability. * *p* < 0.05, *n* = 6. (**B**,**C**) The ECKO mice at two months of age exhibited increased heart weight (HW) to body weight (BW) ratio and decreased fractional shortening. * *p* < 0.05, *n* = 6. (**D**,**E**) H&E-stained heart sections showed marked vascular thrombosis (*arrows*) with extensive inflammatory cell infiltration in the two-month-old ECKO mice. * *p* < 0.05, *n* = 6. Scale bar, 25 µm.

To test for the impact of EC-specific MCPIP deletion on vascular permeability, Evans blue dye (EBD) was injected in the wild type and ECKO mice at two months of age via the tail vein, and various organs (ear, heart, and kidney) were perfused 30 min later with saline to clear the remaining dye from the circulation. Significant EBD accumulation in the extravascular tissues of the ears, heart and kidneys was observed in the ECKO mice (Figure 2A). The ECKO mice also exhibited an increased heart-to-body weight ratio (Figure 2B), which was reflected by the reduced left ventricular fractional shortening with significant left ventricular hypertrophy and dilation, as evidenced by echocardiographic measurements (Figure 2C). Histological analyses of cardiac tissues from ECKO mice showed significant cellular infiltration in the myocardium and vascular thrombosis (*arrows*) (Figure 2D,E). This observation was reflected by the peripheral blood smear (Figure 1E) that revealed the presence of fragmented red blood cells and a significant reduction in platelets (thrombocytopenia). It is likely that loss of MCPIP in ECs contributes to development of a pro-thrombotic milieu, resulting in widespread consumption of coagulation factors and platelets, leading to generalized vascular thrombosis.

**Figure 3:**
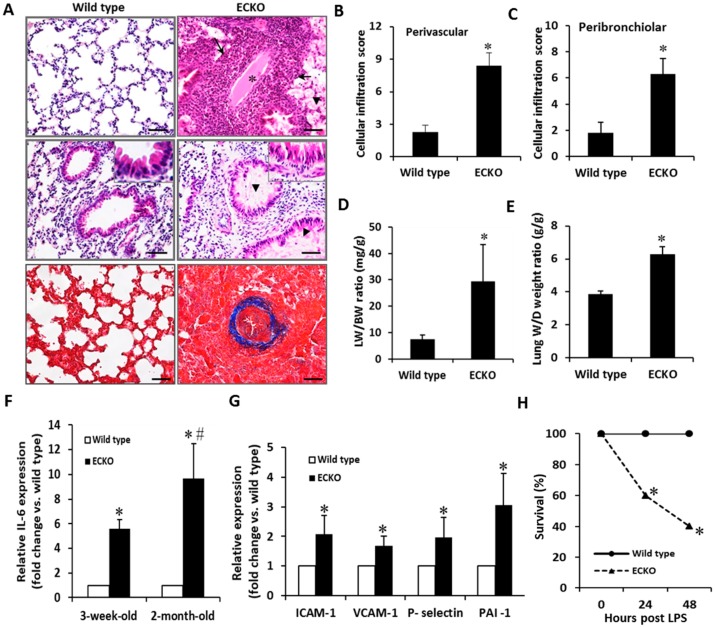
EC-specific MCPIP deletion causes a severe inflammatory response in the lung. (**A**) H&E-stained lung sections showed increased plasma protein leakage (*arrow head*), cellular infiltration (*arrow*), thrombus with luminal obstruction of blood vessels (*star*), perivascular fibrosis (*blue staining*) and diffuse intimal hyperplasia in muscular pulmonary artery in lung tissues of the two-month-old ECKO mice. Scale bar, 25 µm. (**B**,**C**) Both perivascular and peribronchiolar lymphocytic inflammatory infiltrates were markedly increased in lung tissues of the two-month-old ECKO mice. * *p* < 0.05, *n* = 6. (**D**,**E**) The ECKO mice at two months of age showed increased lung wet weight to body weight (LW/BW) ratio and lung wet weight to dry weight ratio. * *p* < 0.05, *n* = 6. (**F**) qRT-PCR analysis of IL-6 expression in lung tissues of the wild type and the ECKO mice at three weeks and two months of age. * *p* < 0.05, ^#^ *p* < 0.01, *n* = 6. (**G**) qRT-PCR analysis of expression of ICAM-1, VCAM-1, P-selectin and PAI-1 in lung tissues of the wild type and the ECKO mice at two months of age. * *p* < 0.05, *n* = 6. (**H**) Survival analysis showed significantly increased mortality of the two-month-old ECKO mice after LPS (25 mg/kg) challenge. * *p* < 0.05, *n* = 10.

The increased red blood cell extravasation, plasma protein leakage, cellular infiltration, and thrombus with luminal obstruction of blood vessels were further documented in the lung tissues of the ECKO mice (Figure 3A). Cellular infiltrates in both perivascular and peribronchiolar lung tissues were markedly increased in the ECKO mice in comparison to the wild type controls (Figure 3B,C), which was reflected by the increased ratio of lung weight to body weight and the ratio of lungs’ wet weight to dry weight (Figure 3D,E). Furthermore, perivascular fibrosis was dominant in the muscular wall of the artery which manifested as severe, diffuse intimal hyperplasia with fibrinoid materials within the blood vessels of the ECKO mice (Figure 3A, *blue* staining). Consistent with these observations, qPCR analyses revealed a marked upregulation of IL-6, a major cytokine that has a diverse role in driving chronic inflammation, EC dysfunction, and fibrogenesis in the lung tissues [20]. IL-6 was elevated in the two-week-old ECKO compared with wild type control mice, and became more pronounced in the two-month-old ECKO mice (Figure 3F). Similarly, the levels of adhesion molecules such as ICAM-1, VCAM-1, E-selectin and PAI-1, markers of EC dysfunction, were markedly increased in the two-month-old ECKO mice compared with the age-matched wild type controls (Figure 3G). Finally, the ECKO mice subjected to LPS (25 mg/kg) challenge showed significantly higher mortality, with the majority of death occurring in the first 24 h after LPS challenge, compared with the wild type controls (Figure 3H). Collectively, these results indicate that EC-specific MCPIP deletion led to compromised vasculature, which manifested as vascular inflammation, thrombosis, myocardial infiltration and failure, pulmonary edema, and high susceptibility to LPS-induced death in mice.

### 2.3. Endothelial-Specific MCPIP Deletion Impairs Tissue Perfusion and Post-Ischemic Angiogenesis

**Figure 4:**
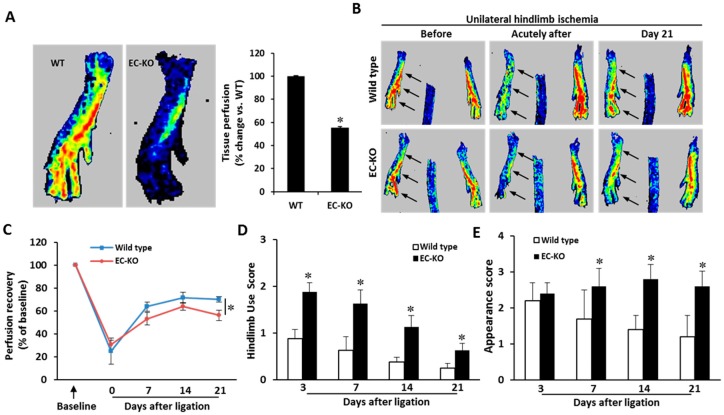
EC-specific MCPIP deletion results in impaired blood perfusion in mice. (**A**) Representative laser Doppler images of plantar foots of the wild type and the ECKO mice at two months of age showed a significant decline in blood perfusion in the ECKO mice. * *p* < 0.05, *n* = 6. (**B**,**C**) Wild type and ECKO mice were subjected to hindlimb ischemia. Blood flow recovery was measured by relative values of foot perfusion between ischemic and non-ischemic legs. Representative laser Doppler perfusion images of plantar foot show decreased perfusion recovery after ligation in the ECKO mice. * *p* < 0.05, *n* = 10. (**D**,**E**) The ECKO mice had worse use score and appearance score. * *p* < 0.05, *n* = 10.

Given that loss of MCPIP in ECs led to compromised vasculature, we assessed the impact of EC-specific MCPIP deletion on vascular function by laser Doppler imaging to assess blood perfusion of the hindlimbs. Compared to the age-matched wild type mice, a significant decrease in blood flow was observed in the two-month-old ECKO mice (Figure 4A). Post-ischemic angiogenesis is critical to limit the ischemic tissue damage and improve blood flow recovery [21]. To determine whether endothelial MCPIP is essential to angiogenesis in vivo, mice were subjected to a hindlimb ischemia and blood flow recovery post ischemia was assayed. After ligation of the femoral artery, an immediate and marked but equal reduction in blood perfusion in the ischemic hindlimb was observed in both the wild type and the ECKO mice (Figure 4B,C). Blood flow recovery in the ischemic hindlimb increased over subsequent days in both the wild type and the ECKO mice; however, blood flow recovery was significantly impaired, which became more apparent 21 days after ligation, in the ECKO mice (Figure 4C). Limb use (index of muscle function) and appearance (index of ischemia) scores were significantly worse in the ECKO mice compared with the wild type controls (Figure 4D, E).

**Figure 5:**
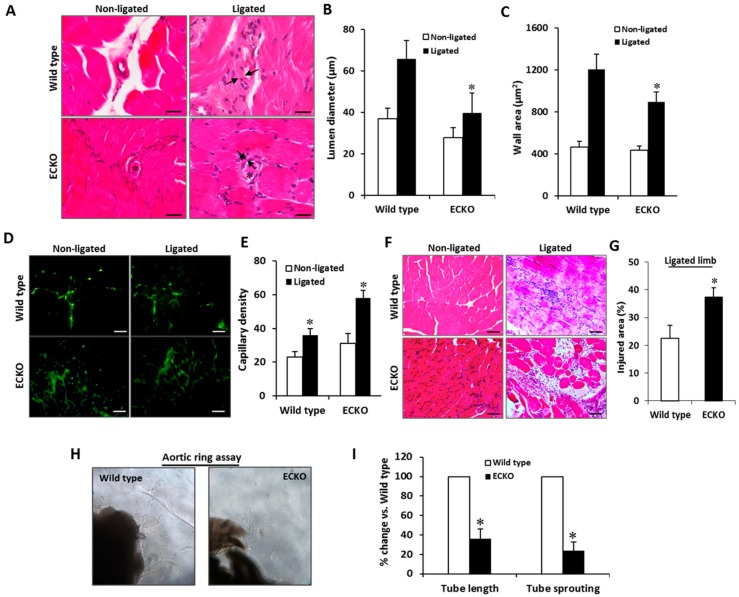
EC-specific MCPIP deletion impairs post-ischemic neovascularization in the ECKO mice. (**A**) Representative H&E-stained sections show collateral remodeling after hindlimb ischemia between the wild type and the ECKO mice at two months of age. *Arrows* indicate collateral wall. *Stars* indicate thrombosis within collateral lumen. Scale bar, 25 µm. (**B**,**C**) The luminal diameter and wall area calculated from the measurements of luminal and perivascular tracing show impaired collateral growth in the ECKO mice. * *p* < 0.05, *n* = 6. (**D**,**E**) Immunostaining with endothelial cell marker CD31 shows increased CD31-positive cells (*green*) in the ischemic muscles of the ECKO mice compared to that seen in the wild type controls at Day 21. Scale bar, 25 µm. * *p* < 0.05, *n* = 6. (**F**,**G**) Representative H&E-stained sections of ischemic muscles show the increased percentage of injury area with cellular infiltration in the ECKO mice. Scale bar, 25 µm. * *p* < 0.05, *n* = 6. (**H**,**I**) Aortas were harvested from the two-month-old ECKO and their littermate controls and cultured in Matrigel for six days. Capillary sprouting and tube length were quantified in six aortic rings under microscopy. * *p* < 0.05, *n* = 6.

Lower blood perfusion in the ECKO mice could indicate impaired post-ischemic angiogenesis. Histomorphometric analysis was performed to examine post-ischemic vascular remodeling. The wild type mice displayed increased collateral diameter and wall area in the ischemic tissues; however, this enhancement was markedly impaired in the ECKO mice (Figure 5A–C). Additionally, capillary density was measured with anti-CD31 antibody in non-ligated and ligated limb tissues by immunofluorescence. The ECKO mice had an increased, morphologically disorganized capillary distribution in comparison with the wild type controls (Figure 5D,E). These data, as well as the reduced blood flow before ligation and impaired recovery of perfusion after ligation, suggest that vasculature in the ECKO mice has an impaired ability to remodel into fully functional vessels due to MCPIP deficiency in ECs. This hypothesis is supported by histological analysis revealing significantly increased areas of necrosis after femoral artery ligation in the ECKO mice when compared with the wild type controls (Figure 5F,G).

We further investigated in vitro tube-forming capacity of ECs from the two-month-old ECKO mice on Matrigel to determine whether an intrinsic functional defect existed. Mouse thoracic aortas were sectioned, embedded in Matrigel, and cultured with endothelial basal medium (Lonza, Allendale, NJ, USA), and neovessel sprouts were blindly counted on Day 6. In contrast to the wild type aortic rings from which microvessels sprouted, aortas from the ECKO mice displayed markedly impaired capillary sprouting and tube elongation (Figure 5H,I), indicating endothelial-specific MCPIP deletion results in impaired angiogenesis.

### 2.4. MCPIP Deficiency Induces NF-κB Activity and Pro-Inflammatory miRNAs in ECs In Vitro and In Vivo

**Figure 6:**
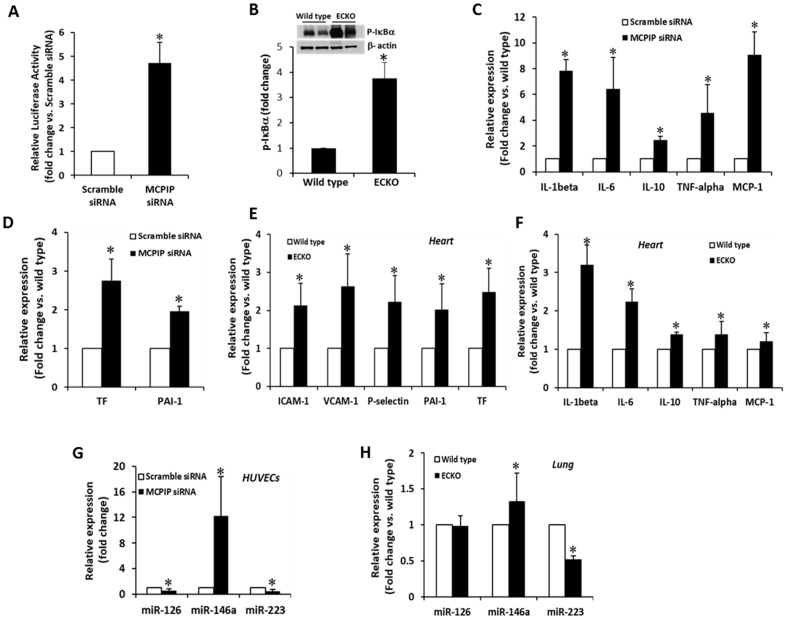
Endogenous MCPIP restrains endothelial activation in vitro and in vivo. (**A**) HUVECs were transfected with a MCPIP specific siRNA or scramble siRNA for 24 h, and the levels of NF-κB activity were assayed by measurement of NF-κB promoter-luciferase reporter activity. Bar graph shows the mean of three independent experiments. * *p* < 0.05. (**B**) Activation of NF-κB in vivo was assessed by measuring the levels of phosphorylated IκBα in the lungs of the ECKO mice and their littermate controls by immunoblots. β-actin was used as a loading control. Bar graph shows pooled data from three animals per group. * *p* < 0.05. (**C**,**D**) The expression of inflammatory genes (IL-1β, IL-6, IL-10, TNF-α, and MCP-1) and pro-coagulants (TF and PAI-1) in the MCPIP-specific siRNA and scramble siRNA-treated HUVECs were assessed by qRT-PCR. Bar graph shows the mean of three independent experiments. * *p* < 0.05. (**E**,**F**) Levels of adhesive molecules (ICAM-1, VCAM-1, and P-selectin), pro-coagulants (TF and PAI-1), and cytokines (IL-1β, IL-6, IL-10, TNF-α, and MCP-1) in the myocardium of the two-month-old ECKO mice and their littermate controls were assessed by qRT-PCR. Bar graph shows pooled data from three animals per group. * *p* < 0.05. (**G**) Knockdown of MCPIP inhibited the expression of miR-126, -223, and increased expression of miR-146a in HUVECs, assayed by qRT-PCR. Bar graph shows the mean of three independent experiments. * *p* < 0.05. (**H**) qRT-PCR analysis of miR-126, -223, and -146a expression in the lung tissue of the two-month-old ECKO mice and their littermate controls. Bar graph represents pooled data from three animals per group. * *p* < 0.05.

MCPIP was reported to regulate inflammatory signaling by suppressing NF-κB activation [10]. We postulated that the constitutive level of MCPIP present in ECs would suppress NF-κB activation and thus keep ECs in a quiescent state. To test this hypothesis, we utilized siRNA knockdown in HUVECs to assess the potential function of the constitutive level of MCPIP in suppressing endothelial activation. Treatment with MCPIP specific siRNA elevated the levels of NF-κB activity by 4.6-fold (Figure 6A). This activation was also reflected by the increased phosphorylation of IκBα in the lung tissues of the ECKO mice (Figure 6B). As expected from NF-κB activation, the mRNA levels of NF-κB-dependent cytokines (e.g., IL-1β, IL-6, MCP-1, and TNF-α) and pro-coagulants (e.g., PAI-1 and TF) were higher in HUVECs transfected with MCPIP specific siRNA than in cells transfected with the scramble siRNA (Figure 6C,D), suggesting activation of HUVECs due to knockdown of MCPIP. To verify the observed effects of MCPIP in vivo on the expression of adhesion molecules and pro-coagulants, heart and lung tissues from the two-month-old ECKO mice and their littermates were examined for expression of ICAM-1, VCAM-1, P-selectin, PAI-1 and TF by real-time quantitative PCR. The mRNA levels of ICAM-1, VCAM-1, P-selectin, PAI-1, and TF in the lung and heart tissues were markedly increased in the ECKO mice compared with their wild type littermates (Figure 6G, E). The levels of mRNA for inflammatory cytokines were also significantly elevated in the myocardium of the ECKO mice when compared to their wild type littermates (Figure 6F).

Several miRs such as miR-126, -146a, and -223 are reported to be highly enriched in ECs, and their dysregulation has been shown to be involved in EC dysfunction [6,7,8]. We assessed the expression of these miRs in HUVECs in response to knockdown of MCPIP, and found that the levels of miR-146a were markedly elevated and that miR-126 and -223 were reduced by knockdown of MCPIP (Figure 6G), suggesting expression of these miRs in HUVECs is regulated by the constitutive level of MCPIP. Next, we assessed the expression of these miRs in the lung tissues of the two-month-old ECKO mice and age- and sex-matched wild type controls. The results showed elevated levels of miR-146a, reduced levels of miR-223, and no change in the levels of miR-126 in the lung tissues of the ECKO mice when compared with their wild type littermates (Figure 6H). Collectively, these results suggest that the constitutive level of MCPIP in ECs may regulate the synthesis of miRs, thus keeping ECs in a quiescent state.

## 3. Discussion

The major finding of this study is that EC-specific MCPIP deletion causes systemic inflammation, vascular leakage, cellular infiltration, microvascular thrombosis, and premature death in mice. These mice also manifested poor blood perfusion and impaired post-ischemic angiogenesis. Mechanically, loss of MCPIP in ECs upregulated NF-κB-dependent pro-inflammatory cytokines and miRs (e.g., miR-146a and -223) associated with pro-inflammatory and pro-thrombotic features. This is the first report providing direct genetic evidence for a critical role of MCPIP in maintaining EC homeostasis and in restraining vascular inflammation.

Endothelial activation is a pro-inflammatory and pro-coagulant state of the endothelial cells lining the lumen of blood vessels. The EC activation process, if uncontrolled, can progress to endothelial dysfunction and vascular inflammation. The presence of EC activation has been demonstrated indirectly by the elevation of circulating levels of soluble adhesion molecules, cytokines and procoagulant molecules [22]. In this study, the pro-inflammatory cytokine levels, which are markers of inflammation, were investigated in mice with EC-specific MCPIP deletion. The pro-inflammatory cytokines (IL-2, IL-3, IL-4, IL-5, IL-6, IL-7, IL-10, IL-12p40, IL-13, IL-17, TNF-α, G-CSF, CCL2, etc.), in serum and lung tissue was found to be higher in the ECKO mice compared to the wild type controls. The increased production of these cytokines and chemokines allows leucocytes to adhere to endothelium and then move into the tissues, leading to vascular inflammation. EC-specific MCPIP knockout mice also showed increased basal levels of circulating adhesion molecules and pro-coagulant molecules such as ICAM-1, VCAM-1, E-selectin and PAI-1, as well as increased cellular infiltration and thrombosis in multiple organs. These results reveal systemic inflammatory characteristics of the ECKO mice due to EC-specific MCPIP deletion and are consistent with the activation of the NF-κB/RNase pathway reported in mice with MCPIP global deletion [10,11], suggesting that endothelial MCPIP is an intrinsic signaling molecule responsible for dampening excessive activation of ECs.

Our current study further supports our general theme that MCPIP functions as a critical cellular suppressor of inflammation in the vasculature. We have previously examined the role of MCPIP in inflammatory responses in several mouse models [23,24,25]. MCPIP global deletion causes systemic inflammation and premature death [10,11]. In a mouse model of middle cerebral artery occlusion (MCAO), MCPIP deficient mice showed significant increase in infarct volume caused by MCAO [25]. Mice with cardiomyocyte-targeted expression of MCPIP protected against endotoxin-induced myocardial inflammation and dysfunction [23]. In a mouse model of myocardial ischemia, cardiomyocyte-targeted expression of MCPIP showed improved survival, decreased cardiac hypertrophy, lower fibrosis and scar formation as well as better left ventricular function after myocardial infarction [24]. These protective effects of MCPIP probably involve its ability to inhibit NF-κB activation as the MCPIP expressing murine hearts showed lower NF-κB signaling. Our findings that loss of MCPIP in ECs in vivo leads to upregulation of inflammatory cytokines, and other NF-κB -dependent mediators (e.g., PAI-1 and TF) suggest that the constitutive activity of MCPIP serves to suppress NF-κB-mediated EC activation and keep ECs in a quiescent state. Supporting this conclusion is the finding that NF-κB inhibitor MG-132 induced MCPIP expression, and knockdown of MCPIP increased NF-κB activity and expression of NF-κB target genes [26]. As further support, knock down MCPIP in ECs increased the phosphorylation of eNOS and NO production, increased expression of VCAM-1 and the adhesion capabilities of THP-1 cells to HUVECs, while overexpression of MCPIP inhibited TNF-α-induced expression of VCAM-1 and THP-1 cell adherence to HUVECs [15].

Several studies have demonstrated the role of miRs in control EC activation [6,7,8]. miR-146a, reported to be enriched in ECs, represses EC activation by inhibiting pro-inflammatory signaling of NF-κB [27]. miR-126, an EC-specific miR, is reported to regulate EC expression of adhesion molecules such as VCAM-1 in HUVECs [28,29]. miR-223 has been shown to inhibit NF-κB activation in ECs and its downregulation promotes glomerular EC activation [30]. Dicer is a critical regulator of the biogenesis of most miRs [31], and a Dicer-independent miR biogenesis pathway that requires Ago catalysis is also reported [32]. MCPIP is previously reported to play a role in regulation of miR biogenesis by suppressing Dicer activity [13]. All three miRs above are highly dependent on Dicer activity [27,28,33], and we found that EC-specific deletion of MCPIP in mice resulted in increased levels of miR-146a, decreased miR-223 and did not affect the expression of miR-126 in the lung tissue. In cultured HUVECs, silencing of MCPIP using siRNA showed similar changes in the expression levels of these three miRs. These results suggest that anti-Dicer activity of MCPIP might participate in keeping ECs in a quiescent state via regulating miR synthesis. The transcription of miRs is also mediated by activation of NF-κB signaling pathway, while some miRs can regulate the formation of other miRs [34]. Thus, up-regulation of miR-146a suggests that endothelial MCPIP deficiency enhances NF-κB activity that drives the expression of miR-146a in activated ECs, which may in turn indirectly regulate the expression of other miRs, such as miR-223. This idea is supported by recent data showing that the expression of miR-146a is downregulated by overexpression of MCPIP in the myocardium [24], and in systemic lupus erythematosus [35]. miR-223 is reported to inhibit ICAM-1 and TF expression in ECs [36,37]. Consistent with these reports is our finding that ECKO mice had decreased expression of miR-223, and increased expression of ICAM-1, VCAM-1 and TF in the heart and lung tissues, suggesting that miR-223 downregulation in MCPIP-deficient ECs may cause EC activation. Taken together, these results suggest that endothelial MCPIP deficiency-mediated dysregulation of endothelial miRs may play a crucial role in endothelial activation.

In endothelial cells, Dicer is constitutively expressed, and Dicer generates miRs that have been shown to impair endothelial differentiation [31] and promote atherosclerosis and vascular inflammation [38]. A role for MCPIP expression in ECs for angiogenesis and vascular integrity is suggested by current findings. Consistent with our previous studies of MCPIP using HUVEC cells, the aortic rings from the ECKO mice displayed impaired capillary sprouting and tube elongation. ECKO mice failed to mount an adaptive angiogenic response, leading to impaired recovery of perfusion and increased necrotic areas after ischemia. In line with this notion, enhanced CD31^+^ cells observed in the tissues of ECKO mice did not constitute functional vessels as indicated by the lack of blood perfusion. It is likely that the constitutive level of MCPIP in ECs provides an important safety net to ensure endothelial differentiation and functional vessel formation. The CD31^+^ cells cannot transdifferentiate into mature endothelial cells needed for functional neo-vascular formation probably due to lack of MCPIP that is known to be required for EC transdifferentiation [17]. On the other hand, although VE-cadherin5-Cre transgenic mice were originally developed as a tool for EC-specific gene deletion, the VEcadherin5 promoter is active in the bone marrow cells and peripheral blood cells [39]. Thus, there is possibility that MCPIP deletion may occur in some hematopoietic lineages such as myeloid cells. As angiogenesis involves monocytic transdifferentiation into endothelial-like cells that is regulated by MCPIP [40], the above cellular defects could contribute to the reduced angiogenesis in ECKO mice in vivo. Thus, the impaired perfusion and impaired post-ischemic flow recovery in the ECKO mice reflect the immature capillary networks and diminished arterial blood flow recovery due to lack of MCPIP in these cells.

**Figure 7:**
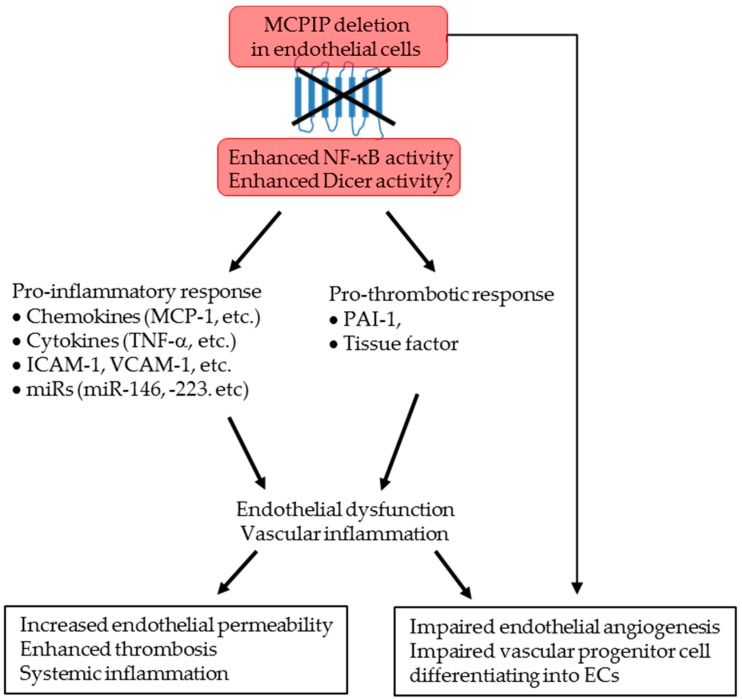
Schematic illustration of phenotype of EC-specific MCPIP knockout mice. Loss of MCPIP in ECs leads to increased endothelial NF-κB and Dicer activities characterized by upregulation of pro-inflammatory and pro-thrombotic mediators, resulting in vascular inflammation, thrombosis, and defects in endothelial angiogenesis, leading to systemic inflammation, impaired vascular integrity, and post-ischemic vascular remodeling in these mutant mice.

In conclusion, the present study with EC-specific MCPIP deletion provides the first in vivo evidence for the role of MCPIP in keeping ECs in a quiescent state. Endothelial MCPIP deletion enhances NF-κB activation and Dicer activity in ECs, resulting in upregulation of adhesion molecules, pro-inflammatory mediators, pro-coagulants, and dysregulation of miRs, leading to the phenotypic defects we describe in the ECKO mice (Figure 7). Our work collectively highlights a previously unrecognized role of MCPIP in control of EC homeostasis and function, and reveals that endothelial MCPIP serves as a key regulator of endothelial biology and that therapeutic elevation of MCPIP in endothelium may represent a novel approach for interfering with EC activation-initiated acute or chronic immune-inflammatory diseases.

## 4. Materials and Methods

### 4.1. EC-Specific MCPIP Deletion in Mice

To specifically inactivate the MCPIP gene in ECs, the floxed MCPIP mice were intercrossed with transgenic mice carrying the Cre recombinase gene under the control of the VE-cadherin-5 promoter promoter [18]. The Cdh5-Cre^+/−^/MCPIP-LoxP^+^ mice were then bred with MCPIP^LoxP/LoxP^ (wild type) mice [19] to generate the Cdh5-Cre^+^/MCPIP^LoxP/LoxP^ (designed as MCPIP endothelium conditional knockout, called ECKO) mice. Both mice were on a C57BL/6J genetic background. Genotyping was performed by polymerase chain reaction (PCR) as described in a previous publication [19]. All animal procedures and protocols used in this study were approved by the University of Central Florida Animal Care and Use Committee (IACUC Number 14-29, approved date: 8 May 2015) in accordance with the Guide for the Care and Use of Laboratory Animals published by the US National Institutes of Health (NIH Publication No. 85-23, revised 2011).

### 4.2. Vascular Permeability Assay In Vivo

Wild type and ECKO mice were anesthetized with inhalation of 1.5 % isoflurane via a nose cone and tail veins were injected with Evans blue dye (Sigma-Aldrich, St. Louis, MO, USA) at 0.1 mL of 0.5% solution/10 g body weight as described. Mice were sacrificed and perfused with heparinized saline to clear any remaining dye in the circulation. Ear, heart, and kidney tissues were excised and weighed. Evans blue extraction was performed by drying the tissue in 60 °C incubator overnight and immersing it in 300 µL of formamide at 60 °C for 24 h. Absorbance values of the extracted dye were measured at 610 nm and normalized to tissue weight (mg).

### 4.3. Preparation of Bone Marrow and Blood Smears

Mice were anesthetized, and heparinized blood was collected from the tail vein. Bone marrow aspiration from mice was performed as described previously [41]. Smears of blood and bone marrow cells were prepared, dried, and fixed in cold methanol.

### 4.4. Histological and Histomorphometric Assessment

Mouse tissues were collected and weighed to establish the organ-to-body weight ratios. Then, tissues were fixed in 10% (*v*/*v* buffered formalin and embedded in paraffin for histological analysis or snap frozen in liquid nitrogen and stored at −80 °C for RNA and protein extraction. Sections from paraffin-embedded tissues were stained with hematoxylin and eosin (H&E). Inflammatory cell infiltration in the tissues was estimated over the total sections of the tissues analyzed, using the inflammation scores as previously described: 0 = normal, 1 = scarce cellular infiltrate, 2 = diffuse infiltrate, 3 = abundant infiltrate and 4 = granulomatous-like infiltrate. To detect fibrosis in lung sections of mice, collagen deposition was visualized by Masson’s Trichrome staining as previously described [24].

### 4.5. Fluorescent Immunohistochemical Analysis

The 5-μm-thick paraffin sections were cut and fluorescent immunohistochemical analysis of sections was performed as previously described [24]. Cell infiltration in the skeletal muscles was examined by using anti-mouse Mac-3 antibody (1:200; Cedarlane Labs, Burlington, ON, Canada) overnight at 4 °C followed by incubation with a Cy3-conjugated secondary antibody (1:500; Chemicon International, Temecula, CA, USA). The slides were then washed in PBS and stained with anti-sarcomeric α-actin antibody (1:200; Abcam Inc. Cambridge, MA, USA) and incubated with an FITC-conjugated secondary antibody (1:500; Chemicon International). The nuclei were stained with DAPI (Vector Laboratories, Burlingame, CA, USA). Capillary density from wild type and ECKO mice was assessed by immunostaining with anti-platelet endothelial cell adhesion molecule-1 (PECAM-1/CD31) antibody (1:200, LifeSpan BioSciences, Nottingham, UK) overnight at 4 °C and then with a FITC-conjugated secondary antibody (Chemicon International). Digital images were captured with a Nikon digital camera mounted directly onto a fluorescence microscopy. Images were analyzed with computerized imaging software (NIS-Elements AR 3.10, Nikon Instruments Inc., Melville, NY, USA) as previously described [24].

### 4.6. RNA Isolation and Quantitative Real-Time RT-PCR (qRT-PCR)

RNA was extracted from tissues using the miRCURYTM RNA isolation kit (Exiqon, Wobum, MA, USA). The isolated RNA was used for miR and mRNA analysis. cDNA was prepared using the Universal MicroRNA cDNA Synthesis Kit II according to the manufacturer’s protocol (Exiqon). qRT-PCR was carried out in a 7500 Fast Real-time PCR system using Fast SYBR^®^ Green Master Mix reagents (Applied Biosystems, Foster, CA, USA). Relative expression of the targeted genes was calculated using the comparative threshold cycle (Ct) method as we described previously [24]. qRT-PCR primers are indicated in Table S1. Primers for Mouse ICAM-1, VCAM-1 and P-selectin were bought from realtimeprimers.com. qRT-PCR analyses for detection of miR-126, -146a, and -223 were performed using ExiLENT SYBR^®^ Green Master Mix reagents (Exiqon). The primers for mature miR-126, -146a, -223, and U6 reference primers were obtained from Exiqon Inc. U6 small nuclear RNA was used as endogenous control for data normalization.

### 4.7. Knockdown Assays with siRNA

HUVECs were transfected with siRNA specific for MCPIP or a negative control scrambled siRNA (Ambion Inc., Austin, TX, USA) with lipofectamine 2000 as previously described [17]. Cellular lysates were isolated 24 h post-transfection to analyze expression of adhesive molecules (ICAM-1, VCAM-1, E-selectin, and P-selectin), pro-coagulation factors (PAI-1 and TF), cytokines (TNF-α, IL-1β, IL-6, and MCP-1), and miRs (miR-126, -146a, and -223) by qRT-PCR. Primers used for qRT-PCR are indicated in Table S1. To analyze NF-κΒ activity, HUVECs were first transfected with siRNA specific for MCPIP or a scrambled siRNA, and after 24 h the cells were transfected with 1 μg of a 5× NF-κB element-luciferase reporter (Promega, Madison, WI, USA) and 100 ng of pRL Renilla luciferase construct (Promega) (for normalization of transfection efficiency). Cellular lysates were isolated 24 h post-transfection using a passive lysis buffer and luciferase activity was monitored using the Dual Luciferase Reporter Assay System (Promega).

### 4.8. Western Blot Analysis

Total proteins were extracted from mice tissues with ice-cold lysis buffer (Roche Applied Sciences, Indianapolis, IN, UAS) as we described in a previous publication [17]. Equal amounts of proteins were run on 4–20% polyacrylamide gels (Mini-Protean TGX; Bio-Rad Laboratories, Hercules, CA, USA), and transferred to nitrocellulose membranes (Bio-Rad Laboratories). After blocking with 4% bovine serum albumin, membranes were incubated with the anti-phospho-IкBα (1:1000; Cell Signaling Technology, Danvers, MA, USA) or polyclonal goat anti-actin (1:5000; Sigma, St. Louis, MO, USA) antibodies. The immune complexes were detected using appropriate peroxidase-labeled secondary antibodies (Santa Cruz Biotechnology, Dallas, USA) and chemiluminescence ECL kit (Amersham™, GE Healthcare Bio-Sciences, Pittsburgh, PA, USA). Specific bands were quantified by densitometry using Alpha imager 2200 software (Genetic Technologies, Inc., Miami, FL, USA).

### 4.9. Ex Vivo Aortic Ring Assay

The thoracic aortas from ECKO and age- and sex-matched wild type mice were freshly isolated and the fat layer and adventitia of aorta were removed. Aortic ring segments approximately 1 mm in length were prepared and individual rings were embedded in 300 μL Matrigel (BD Bioscience, San Jose, CA, USA) polymerized in a 24-well plate for 30 min in 37 °C incubator. EC culture medium (Lonza, Basel, Switzerland) was added and the medium was changed every other day. Neovessel outgrowth was monitored and imaged by phase-contrast microscopy throughout the experiment, and capillary sprouting and tube length were determined and averaged with the use of at least nine segments of aorta from three mice per group.

### 4.10. Hindlimb Ischemia Model and Assessment of Post-Ischemic Functional Recovery

Occlusion of the right femoral artery in ECKO and wild type mice was performed as previously described [42]. Mice were anesthetized and maintained with inhalation of 1.5% isoflurane and 1% oxygen, and hair of inguinal areas was removed with Nair cream. All limbs were secured to a surgical plate on a heated pad (37.0 ± 0.5 °C). A vertical longitudinal incision was made in the right hindlimb, and the right femoral artery and its branches were dissected and ligated with 6-0 nylon sutures (Ethicon Inc., Somerville, NJ, USA), and the arterial segment between the sutures was excised. The skin was closed by interrupted 4-0 sutures (Ethicon Inc., Somerville, NJ, USA). After surgery, the mice were placed in individual cages, and allowed to recover fully from the anesthesia. Animals were visually examined on Days 3, 7, 14, and 21 for hindlimb use score (from 0 for normal to 3 for fragging foot) and/or appearance score (from 0 for normal to 11 for partial atrophy of forefoot) to assess limb ischemia and functional recovery as previously described [43].

### 4.11. Assessment of Blood Perfusion by Laser Doppler Imaging

A laser Doppler imaging system (Moor Instruments Inc., Wilmington, DE, USA) was used to assess hindlimb blood flow by scanning both real paws before, immediately after ligation, and at Days 7, 14, and 21 post-ligation as previously described. Animals were anesthetized and maintained with inhalation of 1.5% isoflurane and 1% oxygen and their body temperature was maintained at 37.0 ± 0.5 °C with a heating pad. Blood flow in the ischemic (ligated) and contralateral non-ischemic (non-ligated) legs in ECKO mice or their corresponding wild type controls were measured simultaneously. Low or no perfusion is shown as dark blue, and the highest perfusion is shown as red. The obtained images were analyzed quantitatively with Moor LDI software, and data were expressed as the ratio of blood flow in the ischemic leg to blood flow in the contralateral non-ischemic leg.

### 4.12. Multiple Analytic Profiling on Serum

Blood samples were drawn from two-month-old male ECKO mice and their corresponding wild type controls. After clotting for 1 h at room temperature, samples were centrifuged at 1000× *g* for 10 min at 4 °C and stored at −80 °C until further analysis. Testing for cytokine, chemokine, and circulating adhesive molecule levels was performed in the laboratory of Eve Technologies using a 32-plex Discovery Assay and a 6-plex Cardio Array (Eve Technologies, Calgary, AB, Canada). Assays were performed in duplicate by following the standard operating protocol provided by Eve Technologies.

### 4.13. Statistical Analysis

Results are expressed as mean ± SD from at least three independent experiments. Statistical analyses were performed using two-tailed Student *t* test between two groups. A *p* value less than 0.05 was defined as statistically significant. All statistical analyses were conducted using GraphPad Prism 7.0 software (San Diego, CA, USA).

## Figures and Tables

**Table 1:** Serum cytokine profiles in the wild type and the ECKO mice.

	Wild Type (*n* =3)	ECKO (*n* = 3)	*p* Value
Cytokines (pg/mL)			
G-CSF	228.7 ± 11.8	707.1 ± 8.2	<0.0001
M-CSF	20.6 ± 9.8	59.1 ± 35.7	0.146
IL-1β	19.5 ± 7.1	30.2 ± 8.2	0.1627
IL-2	19.2 ± 5.9	221.7 ± 18.8	<0.0001
IL-3	6.6 ± 2.8	18.5 ± 0.8	0.002
IL-4	0.2 ± 0.03	3.8.7 ± 0.05	<0.0001
IL-5	5.8 ± 0.8	381.9 ± 8.4	<0.0001
IL-6	1.0 ± 0.7	22.8 ± 0.7	<0.0001
IL-7	8.1 ± 3.3	14.9 ± 1.1	0.027
IL-10	6.6 ± 3.1	54.8 ± 16.3	0.007
IL-12p40	29.8 ± 5.3	71.6 ± 10.7	0.003
IL-13	62.6 ± 22.1	760.2 ± 102.1	0.0003
IL-15	97.1 ± 67.7	127.6 ± 33.2	0.522
IL-17	2.5 ± 0.4	6.0 ± 0.3	0.0003
TNF-α	16.1 ± 1.4	24.9 ± 5.2	0.047
Chemokines (pg/mL)			
CCL11	482.8 ± 72.1	1420.2 ± 126.2	0.0004
CCL2/MCP-1	59.9 ± 6.7	96.6 ± 3.4	0.001
MIP-1α	46.7 ± 38.2	335.3 ± 39.2	0.0008
MIP-1β	11.4 ± 4.1	244.3 ± 40.5	0.0006
CXCL9/MIG	74.4 ± 8.6	143.9 ± 0.9	0.0002
RANTES	43.5 ± 8.1	76.4 ± 28.1	0.123
Othrers (pg/mL)			
KC	83.6 ± 6.5	524.5 ± 23.5	<0.0001
LIX	7302.1 ± 858.9	120 ± 45.3	0.0001
IP-10	59.5 ± 6.7	3720.3 ± 212.3	<0.0001
LIF	1.4 ± 0.9	3.4 ± 0.3	0.021
VEGF	1.0 ± 0.4	6.9 ± 0.4	<0.0001
PAI-1	4.6 ± 0.6	19.7 ± 0.8	<0.0001
sP-selectin	166.8 ± 4.6	226.6 ± 6.9	0.0002

## Supplementary Materials

Supplementary materials can be found at http://www.mdpi.com/1422-0067/20/1/172/s1.

## Abbreviations

MCPIP	MCP-1-induced protein
MCP-1	Monocye chemoattractant protein 1
ECs	Endothelial cells
ECKO	MCPIP endothelium conditional knockout
HUVECs	Human umbilical vein endothelial cells
PCR	Polymerase chain reaction
qRT-PCR	Quantitative reverse transcription-PCR
ICAM-1	Intercellular Adhesion Molecule 1
VCAM-1	Vascular cell adhesion molecule 1
PAI-1	Plasminogen activator inhibitor-1
TF	Tissue factor
TNF-α	Tumor necrosis factor alpha
IL-1β	Interleukin 1 beta
IL-6	Interleukin 6
NF-κB	Nuclear factor kappa-light-chain-enhancer of activated B cells
