# Supplementary material for: Essential Role of Endothelial MCPIP in Vascular Integrity and Post-Ischemic Remodeling
Source: Int J Mol Sci. 2019 Jan 5;20(1):172. doi: 10.3390/ijms20010172 (PMC6337340; doi:10.3390/ijms20010172)
Supplement: Supplementary file 1 [file ijms-20-00172-s001.pdf]

Essential Role of Endothelial MCPIP in Vascular Integrity and Post-Ischemic Remodeling

Zhuqing Jin, Jianli Niu, Nidhi Kapoor, Jian Liang, Edilu Becerra and Pappachan E. Kolattukudy

SupplementaryMaterials:

Table S1. qRT-PCR primers used for measuring gene expression

Mouse β-actin	Forward	5′-CTCTTCCAGCCTTCCTTCCTG-3′
	Reverse	5′-CACCTTCACCGTTCCAGTTT-3′
Mouse IL-1β	Forward	5′-GCCCATCCTCTGTGACTCAT-3′
	Reverse	5′-AGGCCACAGGTATTTTGTCG-3′
Mouse IL-6	Forward	5′-TCGTGGAAATGAGAAAAGAGTTG-3′
	Reverse	5′-AGTGCATCATCGTTGTTCATACA-3′
Mouse TNF-α	Forward	5′-CTGAGGTCAATCTGCCCAAGTAGTAC-3′
	Reverse	5′-CTTCACAGAGCAATGACTCCAAAG-3′
Mouse IL-10	Forward	5′-GGAAGACAATAACTGCACCCACTT-3′
	Reverse	5′-CCGCAGCTCTAGGAGCATGT-3′
Mouse MCP-1	Forward	5′-CCTGGATCGGAACCAAATGA-3′
	Reverse	5′-CCTTAGGGCAGATGCAGTTTTAA-3′
Mouse PAI-1	Forward	5′-GTATGACGTCGTGGAACTGC-3′
	Reverse	5′-TTTCTCAAAGGGTGCAGCGA-3′
Mouse TF	Forward	5′-TCAAGCACGGGAAAGAAAAC-3′
	Reverse	5′-CTGCTTCCTGGGCTATTTTG-3′
Human IL-1β	Forward	5′-GCCAATCTTCATTGCTCA-3′
	Reverse	5′-GAAGGTGCTCAGGTCATTCT-3′
Human IL-6	Forward	5′-GGAGACTTGCCTGGTGAAAA-3′
	Reverse	5′-TGGACTGCAGGAACTCCTTA-3′
Human TNF-α	Forward	5′-GTGCTGGCAACCACTAAGAAT-3′
	Reverse	5′-TTTCACCAGGCAAGTCTCCT-3′
Human IL-10	Forward	5′-GCCAATCTTCATTGCTCAA-3′
	Reverse	5′-GAAGGTGCTCAGGTCATTCT-3′
Human MCP-1	Forward	5′-TCGCTCAGGCAGATGGAATCAATG-3′
	Reverse	5′-AGTTTGGGTTTGCTTGTCCAGGTG-3′
Human ICAM-1	Forward	5′-GCTCAAGTGTCTAAAGGATGGC-3′
	Reverse	5′-CATTATGACTGCGGCTGCTA-3′
Human VCAM -1	Forward	5′-GCTGCTCAGATTGGAGACTCA-3′
	Reverse	5′-CGCTCAGAGGGATGTCTATC-3′
Human E-selectin	Forward	5′-AATCCAGCCAATGGGTTCG-3′
	Reverse	5′-GCTCCCATTAGTTCAAATCCTTCT-3′
Human PAI-1	Forward	5′-TGCTGGTGAATGCCCTCTACT-3′
	Reverse	5′-CGGTCATTCCCAGGTTCTCTA-3′
Human TF	Forward	5′-GCCAGGAGAAAGGGGAAT-3′
	Reverse	5′-CAGTGCAATATAGCATTTGCAGTAGC-3′
Human β-actin	Forward	5′-AGAGCTACGAGCTGCCTGAC-3′
	Reverse	5′-AGCACTGTGTTGGCGTACAG-3′
